# Improved long-term outcomes after heart transplantation utilizing donors with a traumatic mode of brain death

**DOI:** 10.1186/s13019-019-0963-2

**Published:** 2019-07-22

**Authors:** Eilon Ram, Jacob Lavee, Dov Freimark, Elad Maor, Yigal Kassif, Leonid Sternik, Alexander Kogan, Yael Peled

**Affiliations:** 1 Heart Transplantation Unit, Leviev Cardiothoracic and Vascular Center, Sheba Medical Center, Tel Hashomer, Ramat Gan, Israel; 2 Sackler School of Medicine, Tel Aviv University, Tel Aviv, Israel; 3 Department of Cardiac Surgery, Sheba Medical Center, Tel Hashomer, 52621 Ramat Gan, Israel

**Keywords:** Heart transplantation, Mode of brain death, Donor, Recipient

## Abstract

**Background:**

The donor’s mode of brain death (BD), being associated with impairment of myocardial function and hemodynamic performance, impacts the prognosis of the heart transplantation (HTx) recipient.

**Methods:**

All patients who underwent HTx between 1996 and 2017 were categorized according to donor’s BD mechanism: traumatic BD (TBD) versus non-traumatic BD (NTBD).

**Results:**

The TBD group included 105 recipients, and the NTBD group, 85 recipients. Kaplan-Meier survival analysis showed that overall survival was significantly higher for recipients of TBD hearts (10-year survival 58.1 vs. 37.6%, *p* = 0.044). Consistently, multivariate analysis showed that TBD was independently associated with a significant 43% reduction in mortality [95% confidence interval (CI) 0.42–0.75, *p* = 0.033]. Rejection rate was lower in the TBD group (total rejection score 0.44 ± 0.32 vs. 0.51 ± 0.38, *p* = 0.04; any rejection score 0.38 ± 0.26 vs. 0.45 ± 0.31, *p* = 0.030), and freedom from cardiac allograft vasculopathy (CAV) was significantly higher in recipients of traumatic vs. non-traumatic donors (10 years: 82.9 vs. 62.4%, log-rank *p*-value = 0.024). Multivariate analysis showed a significant 42% reduction in CAV [hazard ratio (HR) = 0.58, 95% CI 0.51–0.85, *p* = 0.022).

**Conclusion:**

Mode of brain death significantly impacts HTx outcomes, with TBD being associated with reduced mortality, rejections and CAV.

## Background

In heart transplantation (HTx), proper matching between donor and recipient is of paramount importance. Nonetheless, donor selection remains a complex and controversial topic, with most of the recommendations being based on consensus of opinion [1, 2]. It is thus critical that we, as heart surgeons, constantly review basic assumptions that rely on previous studies, since the ongoing progress in all fields of medicine is eminently evident at the donor-recipient crossroad at which several areas of medicine meet. It is thus not surprising that the criteria that are used for defining the suitability characteristics for donor hearts are constantly being revised [3, 4], particularly in light of the shortage of available donor hearts, combined with increased demand.

The above notwithstanding, brain dead donors remain the main contributors to organ, particularly heart, transplants worldwide [5]. The importance of this statistic lies in the consensus that the mode of brain death (BD) of the donor may influence the recipient’s outcome [6–8]. BD can cause significant heart injury as a result of excessive catecholamine secretion and endocrine and/or hemodynamic disturbances with consequent organ hypoperfusion, resulting in ischemia/reperfusion injury after transplantation [9, 10]. Furthermore, vasopressors will have been administered to > 90% of BD donors [11]. Among the different etiologies that underlie BD, there has been a relative decline in the last decade in the proportion of donors with traumatic brain injury and an increase in those with anoxic brain injury, as reflected in epidemiological data for the distribution of causes of BD in organ donors [12–15].

Even though early results suggest that the mode of donor BD might impact recipient survival and vasculopathy [6, 7], the impact of this factor on HTx outcomes has been poorly studied and quantified. Such data are likely to be of significant importance in today’s era of technological developments and changing criteria for donor characteristics. Therefore, the present study was conducted to quantify the impact of the mode of donor BD on HTx outcomes.

## Methods

### Study population and registry design

Our study population comprised 190 consecutive adult patients (> 18 years of age) transplanted at a single center between July 1996 and July 2017. Data for each patient were systematically recorded upon enrollment in the study and during each subsequent visit or medical contact. Clinical data, recorded on prospectively designed forms, included comprehensive information regarding the transplantation procedure, immunosuppression, occurrence of major cardiac events, and treatment during long-term follow-up. Donors’ data were obtained from the National Organ Transplantation Center and from the records of the hospitals at which the donors had died. The study was approved by our institutional review board.

### Definitions of brain death mechanism

Brain death diagnosis was determined in accordance with clinical guideline for physicians regulated by the Ministry of Health, followed by the Brain Respiratory Death Act which was established in 2008. HTx patients were divided into two groups based on the mechanism of donor BD, namely, traumatic BD (TBD) vs. non-traumatic BD (NTBD) [6, 7], where TBD was defined as BD resulting from a blunt and penetrating head trauma (e.g., gunshot wound to the head, accidental head trauma) or a clearly definable intracranial bleed that progressed rapidly to brain death [6].

### Immunosuppression

All patients were treated with a triple-drug maintenance immunosuppression regimen, comprising steroids, an antimetabolite, and a calcineurin inhibitor. Conversion to everolimus was based on the patient’s risk profile. All patients received induction therapy with anti-thymocyte globulin.

### Rejections, surveillance and classification

Rejections were diagnosed by routine institutional follow-up or clinically indicated endomyocardial biopsy (EMB), and were classified according to the revised International Society of Heart and Lung Transplantation (ISHLT) classification system for rejection [16]. Routine EMBs were performed every week for the first 4 weeks after HTx, twice a month in the second and third months, once a month for the next 3 months, and thereafter every 3 months until the end of the first year. From the end of the first year until the end of the fifth year, biopsies were performed annually. Total rejection score (TRS), which reflected the severity of the rejection, and any rejection score (ARS), which represented the total number of rejections regardless of severity, were calculated for each patient as follows: TRS as 0R = 0, 1R = 1, 2R = 2, and 3R = 3, and ARS, as 0R = 0, 1R = 1, 2R = 1, 3R =1. All scores were normalized by dividing the cumulative scores by the total number of biopsy specimens taken for each patient throughout the study period [17].

### Cardiac allograft vasculopathy and primary graft dysfunction

The institutional post-transplant care protocol includes annual invasive coronary angiography for the first 5 years following HTx, and thereafter every other year, along with echocardiogram and right heart catheterization. Cardiac allograft vasculopathy (CAV), diagnosed by coronary angiography, and invasive hemodynamic assessment, along with clinical assessment and echocardiography, combined according to the recommended ISHLT standardized nomenclature for CAV [18]. Primary graft dysfunction (PGD) was restricted to 24 h post-surgery and was based on echocardiographic and/or hemodynamic assessment, according to the ISHLT consensus conference recommendations [19].

### Outcome measures

The primary end-point was all-cause mortality. Mortality data were obtained from the Population Registry of the State of Israel, where all deaths are registered, as required by law. Secondary endpoints were: 1) rejections 2) freedom from CAV; and 3) freedom from non-fatal major adverse cardiac events (NF-MACE), defined as the development of acute myocardial infarction, congestive heart failure, stroke, new-onset peripheral vascular disease or the need for percutaneous coronary intervention or implantable cardiac defibrillation.

### Statistical analysis

Descriptive statistics were produced using means and standard deviations for continuous variables (e.g. age), and frequencies for categorical variables (e.g., gender). To examine differences between groups in continuous variables, Mann-Whitney procedures were used to avoid bias for non-normal distributions. To examine differences between groups in categorical variables, Chi-Square tests were conducted. The Kaplan–Meier estimator was used to assess the time to the first occurrence of each endpoint by BD mechanism, and groups were compared using the log-rank test. Multivariable Cox proportional hazard regression analysis was used to evaluate the association between the BD mechanism and the first occurrence of endpoints during follow-up. Covariates included in the multivariate models were identified using the best subset procedure among variables that were predictive of the endpoint and were unbalanced between the two groups; candidate covariates are listed in Tables 1 and 2. Due to significant differences between groups, the following variables were statistically controlled in further analyses: donor’s age and height, hospitalization length, cardiopulmonary resuscitation and hemodynamics on presentation.

**Table 1:** Recipients’ characteristics

	Non-traumatic brain death (*N* = 85)	Traumatic brain death (*N* = 105)	*p*-value
Gender (male, %)	75.4	83.5	0.217
Age (years)	47.4 ± 14.7	46.9 ± 13.9	0.331
Weight (kg)	72.0 ± 19.1	73.6 ± 15.1	0.717
Height (cm)	165.5 ± 27.1	170.9 ± 12.2	0.725
BMI	24.3 ± 4.4	24.8 ± 4.6	0.578
Ischemic etiology for HTx (%)	49.2	54.1	0.622
Hypertension (%)	20.0	27.6	0.221
Diabetes pre (%)	18.5	18.8	0.955
Dyslipidemia (%)	37.9	44.3	0.405
Past smoker (%)	38.2	35.6	0.826
CMV positive serology (%)	91.2	93.6	0.742
Status prior to transplantation (%)			0.228
Status 1	70.8	60.0	
Status 2	29.2	40.0	
Creatinine (mg/dL)	1.4 ± 1.1	1.2 ± 0.5	0.209
Bilirubin (mg/dL)	1.2 ± 0.8	1.1 ± 0.6	0.234
Systolic blood pressure (mmHg)	128.1 ± 22.1	129.1 ± 11.0	0.133
Diastolic blood pressure (mmHg)	73.2 ± 12.8	75.2 ± 11.6	0.257
Systolic PAP (mmHg)	54.1 ± 19.3	49.1 ± 20.1	0.405
Diastolic PAP (mmHg)	26.3 ± 10.8	24.4 ± 11.3	0.883
Mean PAP (mmHg)	36.7 ± 14.5	34.9 ± 13.9	0.994
PCWP	25.4 ± 11.3	25.1 ± 11.2	0.712
CO	3.5 ± 1.1	3.5 ± 1.1	0.671
PVR	3.1 ± 2.0	2.9 ± 2.5	0.871
ICD (%)	35.9	36.1	0.979
LVAD bridge to HTx	23.7	17.6	0.410
PRA > 30%	1.6	3.1	0.351
CMV mismatch	46.2	36.5	0.245
Blood type			0.713
A	46.8	45.8	
AB	8.6	16.3	
B	13.3	22.4	
O	26.9	19.4	

*BMI* Body mass index, *HTx* Heart transplantation, *CMV* Cytomegalovirus, *PAP* Pulmonary artery pressure, *PCWP* Pulmonary capillary wedge pressure, *CO* Cardiac output, *PVR* Pulmonary vascular resistance, *ICD* Implantable cardioverter defibrillator, *LVAD* Left ventricular assist device, *PRA* Panel reactive antibody

**Table 2:** Donors’ characteristics

	Non-traumatic brain death (*N* = 85)	Traumatic brain death (*N* = 105)	*p*-value
Gender [male, %]	74.1	79.7	0.124
Age (years)	37.4 ± 13.1	27.8 ± 10.9	< 0.001
Weight (kg)	74.9 ± 19.5	74.8 ± 15.2	0.106
Height (cm)	170.4 ± 26.7	175.3 ± 10.1	0.037
BMI	25.2 ± 5.2	24.1 ± 3.4	0.118
CMV positive serology (%)	75.0	76.5	0.832
Brain injury interval hours	131.9 ± 86.2	86.3 ± 45.6	0.001
Brain death interval	13.1 ± 3.7	12.6 ± 4.7	0.544
Hospitalization days	5.7 ± 2.3	3.9 ± 2.1	< 0.001
Amines (%)	79.2	77.5	0.712
Amines more > 2 (%)	34.1	42.3	0.312
Treated with thyroxine (T4) (%)	12.8	16.2	0.527
Cardiopulmonary resuscitation	22.5	5.8	0.005
Systolic blood pressure on admission (mmHg)	140.7 ± 38.9	121.4 ± 34.1	0.002
Diastolic blood pressure on admission (mmHg)	85.3 ± 25.8	69.2 ± 20.4	< 0.001
Last systolic blood pressure (mmHg)	125.2 ± 18.2	123.4 ± 8.8	0.512
Last diastolic blood pressure (mmHg)	77.2 ± 9.2	79.2 ± 10.1	0.327

*BMI* Body mass index, *CMV* Cytomegalovirus

To validate our findings, we calculated a propensity score using binary logistic regression. We included the covariates that were found to be significantly different according to Tables 1, 2, 3 (donor’s age, hospitalization length, cardio-pulmonary resuscitation and blood pressure on admission). Data were analyzed with SPSS software version 23. A two-sided 0.05 significance level was used for hypothesis testing.

**Table 3:** Operative and post-operative data

	Non-traumatic brain death (*N* = 85)	Traumatic brain death (*N* = 105)	*p*-value
Operative data			
Ischemic time (minutes)	157.1 ± 42.4	155.4 ± 43.2	0.815
Days from admission to discharge	81.6 ± 12.9	76.3 ± 10.7	0.429
Days from transplant to discharge	33.8 ± 8.6	43.2 ± 16.1	0.679
Early post-operative data			
In-hospital mortality (%)	18.2	15.2	0.101
Primary graft dysfunction (%)	21.5	20.2	0.842
Late post-operative data			
End stage renal failure (%)	7.7	11.0	0.580
Statin post HTx (%)	89.1	92.2	0.446
LDL after HTx (mg/dl)	112.8 ± 34.9	111.5 ± 32.1	0.990
Hypertension after HTx (%)	67.3	65.1	0.927
Diabetes mellitus after HTx (%)	36.4	35.3	0.926
Clinical CMV disease (%)	19.1	19.7	0.919
Follow up (years)	8.6 ± 4.2	7.8 ± 4.8	0.811
Era: after year 2000	56.6	66.7	0.173

*HTx* Heart transplantation, *LDL* Low density lipoprotein, CMV Cytomegalovirus

## Results

### Patient characteristics and early results

The current analysis was based on 190 patients, who were classified into two groups according to donor’s BD mechanism: TBD (*n* = 105) and NTBD (*n* = 85). Baseline clinical characteristics of the study patients by donor’s BD mechanism are shown in Table 1. Recipients’ baseline characteristics were similar in the two groups in terms of age, gender, pre-existing cardiovascular risk factors and hemodynamics prior to the HTx. Donors’ characteristics, according to TBD and NTBD, are shown in Table 2. For both TBD and NTBD groups, donors were younger than recipients and most were males. Donors of the TBD group were 10 years younger than those in the NTBD group, and were characterized by shorter hospitalization prior to donation, shorter brain injury interval until BD determination, reduced incidence of cardio-pulmonic resuscitation, and lower blood pressure on admission. Early in-hospital outcomes were similar between the TBD and NTBD groups, respectively, as follows: in-hospital mortality 15.2 vs. 18.2%, *p* = 0.102; PGD 20.2 vs. 21.5%, *p* = 0.842; and length of hospitalization 21.8 ± 14.2 vs. 23.1 ± 7.6 days, *p* = 0.721 (Table 3). The average follow-up of the groups was similar.

### Mortality

**Fig. 1:**
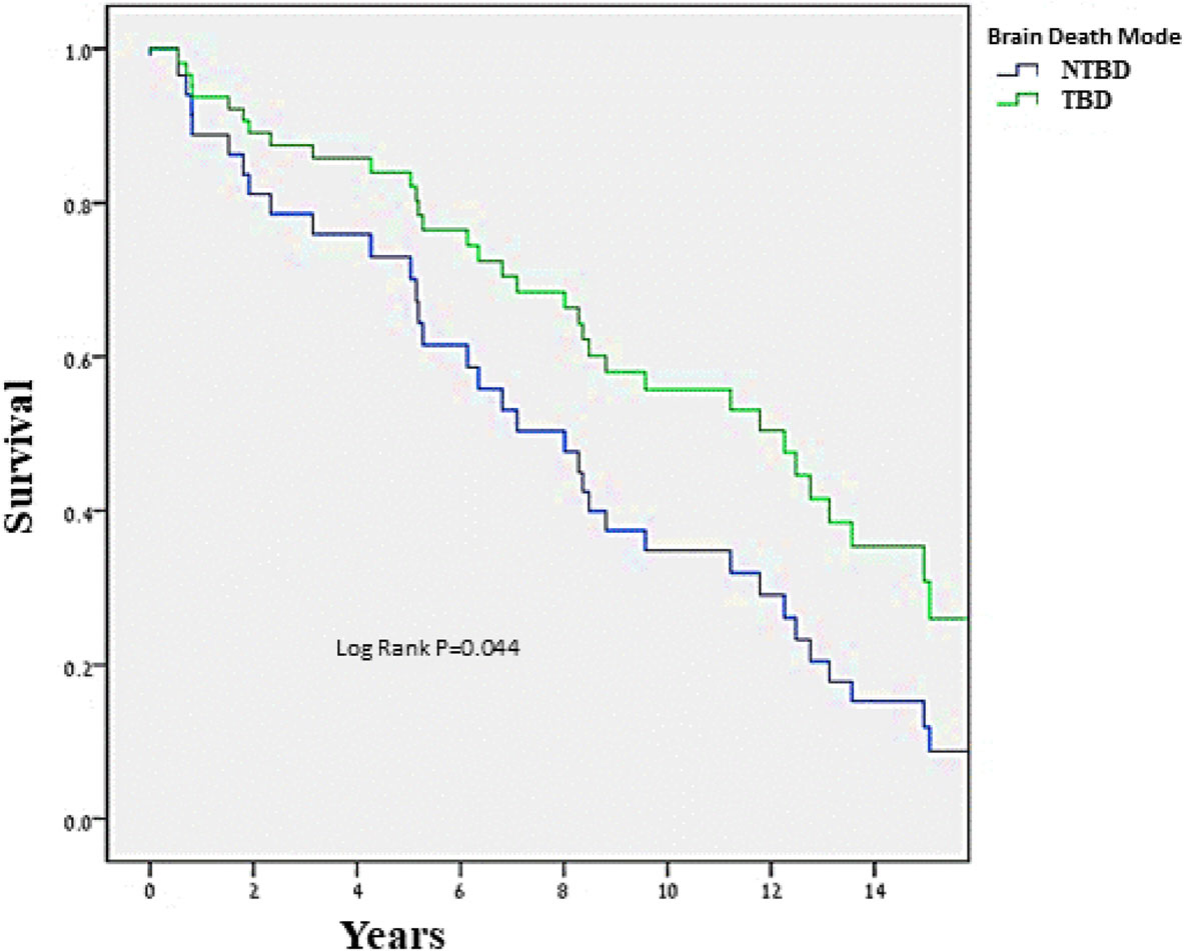
Kaplan–Meier curve of survival by traumatic vs. non-traumatic brain death

Kaplan-Meier survival analysis showed that overall survival was significantly higher for the TBD vs. the NTBD group: At 10 years of follow-up, the rates of survival were 58.1% in the TBD group and 37.6% in the NTBD group (log-rank *p*-value = 0.044 for the comparison between the two groups during follow-up) (Fig. 1). Consistently, multivariate analysis showed that TBD was independently associated with a significant 43% reduction in mortality [hazard ratio (HR) = 0.57, 95% confidence interval (CI) 0.42–0.75, *p* = 0.033; Table 4]. These findings were further validated by propensity score analysis (HR = 0.58, 95% CI 0.44–0.77, *p* = 0.050, Table 4). In addition, it was found that higher donor blood pressure on admission was associated with lower recipient mortality (Systolic: HR = 0.97, 95% CI 0.92–0.98, *p* = 0.042; Diastolic: HR = 0.94, 95% CI 0.89–0.96, *p* = 0.029).

**Table 4:** Adjusted COX model for mortality

	Multivariate analysis			Propensity		
	HR	95% CI	*p-*value	HR	95% CI	*p-*value
Traumatic brain death	0.57	0.42–0.75	0.033	0.58	0.44–0.77	0.050
Donor age	0.91	0.96–1.13	0.921			
Donor height	1.02	0.95–1.13	0.443			
Donor’s hospitalization days	1.02	0.92–1.10	0.693			
Donor CPR	0.99	0.95–1.15	0.976			
Donor systolic blood pressure on admission	0.97	0.92–0.98	0.042			
Donor diastolic blood pressure on admission	0.94	0.89–0.96	0.029			

*HR* Hazard ratio, *CI* Confidence interval, *CPR* Cardiopulmonary resuscitation

### Rejections, CAV and NF-MACE

**Fig. 2:**
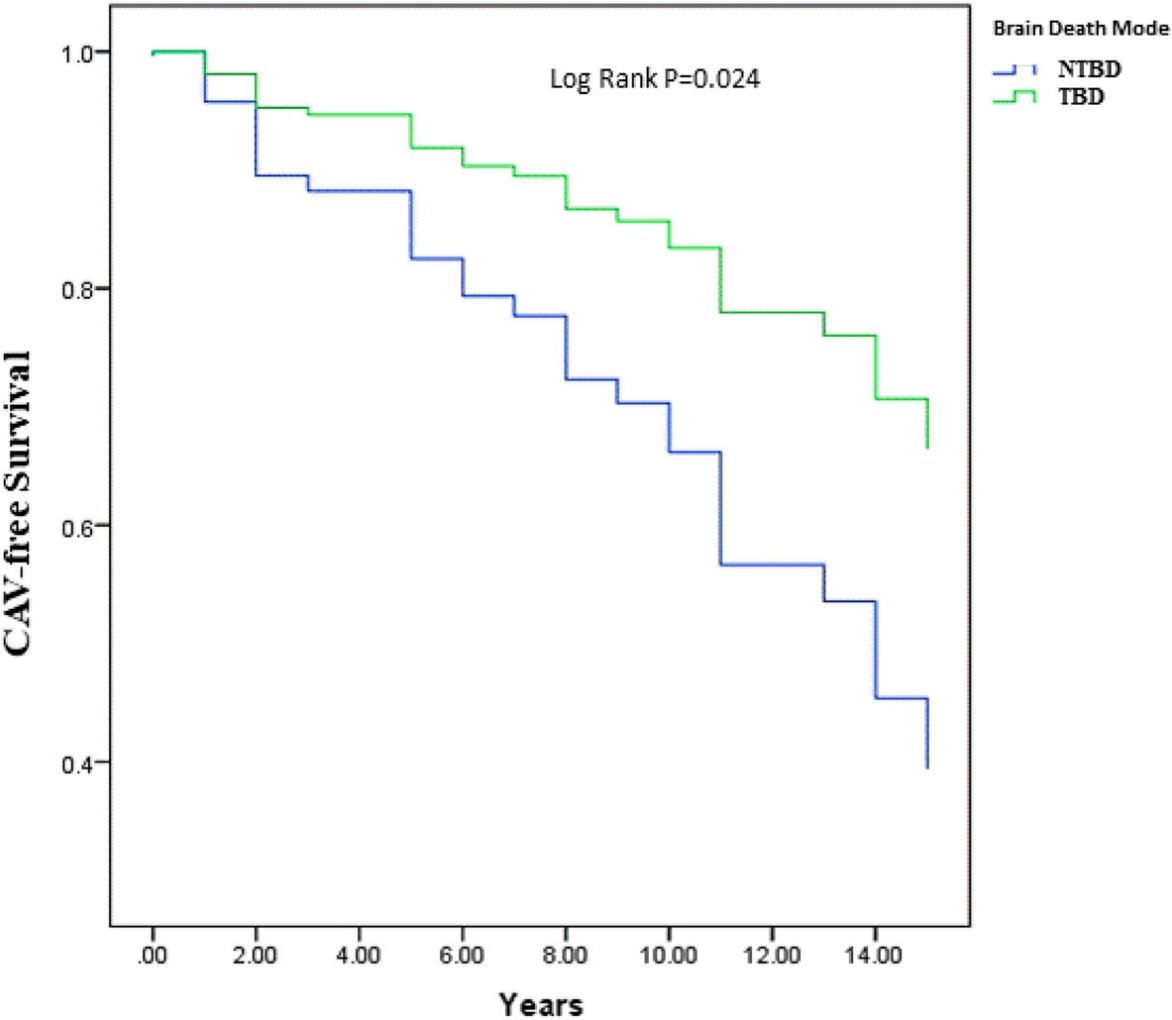
Kaplan–Meier curve of CAV-free survival by traumatic vs. non-traumatic brain death

Recipients of hearts from TBD donors had lower TRS (reflecting the severity of rejection) and ARS (representing the total number of rejections, regardless of severity) than recipients from NTBD donors (TRS: 0.44 ± 0.32 vs. 0.51 ± 0.39, respectively, *p* = 0.040; ARS: 0.38 ± 0.26 vs. 0.45 ± 0.31, respectively, *p* = 0.030). Kaplan-Meier analysis showed that freedom from CAV was significantly higher in recipients of TBD hearts compared with recipients of NTBD organs (10 years: 82.9 vs. 62.4%, log-rank *p*-value = 0.024; Fig. 2). Consistently, multivariate analysis showed that TBD was independently associated with a significant 42% reduction in CAV (HR = 0.58, 95% CI 0.51–0.85, *p* = 0.022; Table 5). These findings were further validated by propensity score analysis (HR = 0.57, 95% CI 0.51–0.81, *p* = 0.025; Table 5). There was no significant difference in 10-year freedom from NF-MACE between the groups (22.9 vs. 27.1%, log rank *p* = 0.553, for TBD vs. NTBD, respectively).

**Table 5:** Adjusted COX model for cardiac allograft vasculopathy

	Multivariate analysis			Propensity		
	HR	95% CI	*p-*value	HR	95% CI	*p-*value
Traumatic brain death	0.58	0.51–0.85	0.022	0.57	0.51–0.81	0.025
Donor age	0.92	0.95–1.11	0.854			
Donor height	1.03	0.85–1.23	0.565			
Donor’s hospitalization days	1.02	0.82–1.26	0.702			
Donor CPR	0.98	0.85–1.25	0.781			
Donor systolic blood pressure on admission	0.98	0.85–1.1	0.891			
Donor diastolic blood pressure on admission	0.93	0.56–1.33	0.341			

*HR* Hazard ratio, *CI* Confidence interval, *CPR* Cardiopulmonary resuscitation

## Discussion

It has been postulated that the “ideal” donor would be a young healthy individual with no pre-existing comorbidities who died as a result of an isolated head trauma [20]. Nonetheless, animal and human post-mortem studies focusing on brain death due to a rapid increase in intracranial pressure have not provided unequivocal validation of this premise [9, 21–23]. An analysis of our results, which serve to expand the limited and somewhat scattered literature regarding the effect of mode of donor BD on outcomes of HTx patients, reveals some new – and at times controversial – findings, as discussed below. In summary, we showed that: 1) late mortality is affected by the mechanism of donor BD, with a survival advantage for recipients from TBD donors; 2) TRS and ARS are influenced by donor events that precede the actual engraftment process and are negatively affected by NTBD; 3) the incidence of CAV is significantly lower in recipients of TBD donors; and 4) early outcomes, including PGD and in-hospital mortality, are not influenced by the mechanism of donor BD.

A review of the general transplantation literature reveals that graft function is inferior in kidney transplants from BD donors compared with live donors [24, 25]. Similarly, heart recipients from live donors (domino hearts) show decreased incidence and severity of transplant-associated coronary artery disease compared with recipients from dead donors [26]. BD induces significant systemic derangements, including hemodynamic instability and the release of proinflammatory cytokines, causing significant injury to the organ to be transplanted before procurement. BD also causes a rise in serum catecholamines, which may result in coronary vasoconstriction and myocardial ischemia. In addition, a spinal shock phase may occur, resulting in severe hypotension and hence requiring the use of inotropes and vasopressors to maintain organ perfusion [27, 28]. Indeed, regardless of the BD mechanism, most of the donors in this study were supported with inotropes, with approximately 40% being supported by combination of more than two inotropes. The types and doses of inotropes and hormones were similar, irrespective of the cause of BD, and the resulting systolic and diastolic blood pressures were similar prior to procurement, despite the facts that NTBD donors presented with higher systolic and diastolic blood pressure on admission and cardiopulmonary resuscitation (CPR) was necessitated for more patients of this group.

In the current study, we showed a survival advantage and a lower incidence of CAV for recipients from TBD donors. Our results thus appear to contradict the seminal pioneering study of Mehra et al. [6], who evaluated, by intravascular ultrasound (IVUS), the coronary arteries of 61 consecutive HTx recipients between 1993 and 1995, according to ‘explosive’ (equivalent to ‘traumatic’ in our study) vs ‘non-explosive’ BD donors. They showed that HTx recipients with allografts from explosive BD donors demonstrated increased maximal intimal thickness of the arteries and more cardiac events and hence lower survival. Their IVUS study was performed at an average of 2.4 years post HTx, with a follow up of an average of 4 years after the index ultrasound. In our study, CAV assessment in did not include IVUS, so we are not able to make a direct comparison. Moreover, in our study, the survival curves diverged after more than 2 years, with more than 90% of the recipients of TBD hearts currently being treated with statins and having an average LDL of 111 mg/dl (vs 30% and 140 mg/dl, respectively, in the study of Mehra et al. [6]). In general, comparisons are problematic because patient and donor characteristics are significantly different among various studies in terms of ischemic time, donor age, and immunosuppressive protocols. Nonetheless, the findings of the current study complement those of similar studies reporting major discoveries and significant progress in the field [29, 30].

The importance of the nature of the donor brain injury lies in its effect on the donor heart. Traumatic injury to the brain is accompanied by an acute increase in intracranial pressure, which was shown in experimental models to produce more severe hemodynamic alterations and myocardial collapse than other modes of brain injury (due to a steeper rise in epinephrine levels and irreversible myocardial damage secondary to catecholamine-mediated toxicity) [21, 31–34]. In contrast, it had previously been shown that despite BD-associated hemodynamic deterioration in vivo, after explantation and ex vivo assessment, the myocardial function of hearts removed from BD and sham-operated dogs was not significantly different [35]. In an in situ isolated perfused heart model, it was demonstrated that if coronary perfusion pressure was de-coupled from aortic pressure and elevated to pre-brain death levels, coronary blood flow and myocardial contractility were also restored to baseline levels [36]. The above findings imply that cardiac dysfunction after BD is reversible and that changes in donor myocardial function may reflect altered loading conditions and coronary perfusion rather than irreversible injury due to an initial Cushing-type reaction and subsequent hormone depletion. Thus, it has been suggested that donor hearts should be carefully evaluated by load-independent indices of cardiac function and that the normalization of loading conditions in the BD donor may lead to an improvement of cardiac performance. An increasing number of clinical studies have indeed demonstrated that cardiac dysfunction can be reversed in potential organ donors and that the clinical outcome of transplant patients receiving hearts from primary marginal donors is comparable to those of normal donors [35–38]. Our results are in accord with these findings.

Mode of brain death significantly impacts long term HTx outcomes, while early outcomes, including PGD and in-hospital mortality, are not influenced by the mechanism of donor BD. Brain death leads to dramatic changes in loading conditions in a decrease of contractile function via the Arnep and garden-hose effects as well as the Frank-Starling mechanism [38–40]. These physiologic regulatory mechanisms give a plausible explanation for decreased cardiac function, without the necessity of irreversible tissue damage or “true” dysfunction. However, early abnormalities (i.e. abnormal plasma catecholamines values and intracellular calcium handling) may eventually lead to endothelial damage, a primary precipitating event in CAV pathogenesis, a major cause of long-term mortality following HTx.

The impressive advance in mechanical circulatory support devices has not yet resulted in a true alternative to HTx. Thus, while HTx will continue to be the gold standard treatment for end-stage heart failure and donors will continue to change in terms of causes of BD toward a non-traumatic etiology [4], HTx surgery will continue to be significantly more challenging with longer and more complex surgeries and increased ischemia times for organs that were previously considered to be borderline or non-transplantable. Nonetheless, despite the increasing complexity of recipients and donors, results of HTx have improved progressively. It is therefore of particular importance to continue research aimed at defining and characterizing the factors that may affect the results of HTx. Once we will have defined the factors associated with less favourable outcomes, the next step will be to find the correct donor-recipient matching parameter that will neutralize the negative influence in the most significant way. An example is a study showing that differences in donor–recipient predicted heart mass modulated the survival associated with donor–recipient sex mismatch [41]. Redistributing organs to recipients most likely to derive maximal benefit will result in expanded organ allocation potential, underscoring the importance of the current study. It is our hope that our findings will contribute to targeting novel strategies aiming to optimize graft preservation, including donation after circulatory determined death and ex-vivo perfusion of hearts [42].

### Study limitations

The major limitation of our study lies in its observational nature. Not all possible confounders were recorded or adjusted for this single-center study with relatively small sample size. No measurements of specific biological markers of BD in the donors were made. Therefore, we could not evaluate objectively the extent of the brain damage and the correlation with recipient outcomes.

## Conclusions

The modality of donor BD significantly influences long-term outcomes after HTx: TBD is associated with reduced mortality, CAV, and a lower rejection score. The mode of BD does not affect early HTx outcomes, including PGD and in-hospital mortality. We conclude by emphasizing that the results of our study do not argue against utilizing NTBD donors but rather represent opportunities for greater utilization of these donors. Survival prediction models should incorporate the BD mechanism with the aim to further optimize matching of donor and recipient. Additional prospective studies are warranted to better delineate the pathophysiologic consequences of BD. The effects of donor and recipient treatment protocols in addition to organ-specific aspects of injury and repair still need to be assessed.

## Data Availability

The datasets used and/or analyzed during the current study are available from the corresponding author on reasonable request.

## Abbreviations

ARS: Any rejection score
BD: Brain death
CAV: Cardiac allograft vasculopathy
CI: Confidence interval
CPR: Cardiopulmonary resuscitation
EMB: Endomyocardial biopsy
HR: Hazard ratio
HTx: Heart transplantation
ISHLT: International Society of Heart and Lung Transplantation
IVUS: Intravascular ultrasound
NF-MACE: Non-fatal major adverse cardiac events
NTBD: Non-traumatic brain death
PGD: Primary graft dysfunction
TBD: Traumatic brain death
TRS: Total rejection score
